# Dog breeds and conformations predisposed to osteosarcoma in the UK: a VetCompass study

**DOI:** 10.1186/s40575-023-00131-2

**Published:** 2023-06-27

**Authors:** Dan G. O’Neill, Grace L. Edmunds, Jade Urquhart-Gilmore, David B. Church, Lynda Rutherford, Matthew J. Smalley, Dave C. Brodbelt

**Affiliations:** 1 Pathobiology and Population Sciences, The Royal Veterinary College, Hawkshead Lane, North Mymms, Hatfield, AL9 7TA Herts UK; 2 School of Veterinary Sciences, University of Bristol and Langford Vets, Stock Lane, Langford, BS40 5DU UK; 3 Clinical Science and Services, The Royal Veterinary College, Hawkshead Lane, North Mymms, Hatfield, Herts AL9 7TA UK; 4 European Cancer Stem Cell Research Institute, School of Biosciences, Cardiff University, Hadyn Ellis Building, Maindy Road, Cardiff, CF24 4HQ UK

**Keywords:** Bone tumour, Bone cancer, Breed, Electronic patient record, Epidemiology, Primary-care, Pedigree, Purebred, VetCompass, Veterinary

## Abstract

**Background:**

Osteosarcoma is a malignant bone neoplasia that has high welfare consequences for affected dogs. Awareness of breed and canine conformational risk factors for osteosarcoma can assist with earlier diagnosis and improved clinical management. Study of osteosarcoma in dogs also offers translational value for humans. Anonymised clinical data within VetCompass on dogs under primary veterinary care in the UK were searched for osteosarcoma cases. Descriptive statistics reported overall and breed-specific prevalence. Risk factor analysis used multivariable logistic regression modelling.

**Results:**

From 905,552 study dogs, 331 osteosarcoma cases were confirmed yielding a one-year period prevalence of 0.037% (95% CI: 0.033–0.041). Breeds with the highest annual prevalence were the Scottish Deerhound (3.28%, 95% CI 0.90–8.18), Leonberger (1.48%, 95% CI 0.41- 3.75), Great Dane (0.87%, 95% CI 0.43- 1.55) and Rottweiler (0.84%, 95% CI 0.64–1.07). The median age at diagnosis was 9.64 years (IQR: 7.97–11.41).

Following multivariable modelling, 11 breeds showed increased odds of osteosarcoma compared with crossbred dogs. Breeds with the highest odds included Scottish Deerhound (OR 118.40, 95% CI 41.12–340.95), Leonberger (OR 55.79, 95% CI 19.68–158.15), Great Dane (OR 34.24, 95% CI 17.81–65.83) and Rottweiler (OR 26.67, 95% CI 18.57–38.29). Compared with breeds with mesocephalic skull conformation, breeds with dolichocephalic skull conformation (OR 2.72, 95% CI 2.06–3.58) had increased odds while breeds with brachycephalic skull conformation showed reduced odds (OR 0.50, 95% CI 0.32–0.80). Chondrodystrophic breeds had 0.10 times the odds (95% CI 0.06–0.15) compared with non-chondrodystrophic breeds. Increasing adult bodyweight was associated with increasing odds of osteosarcoma.

**Conclusions:**

The current study cements the concept that breed, bodyweight and longer leg or longer skull length are all strong risk factors for osteosarcoma in dogs. With this awareness, veterinarians can update their clinical suspicion and judgement, breeders can select towards lower-risk animals, and researchers can robustly define more useful study populations for fundamental and translational bioscience.

**Supplementary Information:**

The online version contains supplementary material available at 10.1186/s40575-023-00131-2.

## Abstract (plain language summary)

Osteosarcoma describes a serious bone tumour. Affected dogs often show a bony or soft tissue swelling with severe pain. Canine and human osteosarcoma show similar patient characteristics, clinical course and tumour biology that make studies on canine osteosarcoma highly valuable to inform on the human disease. This study aimed to interrogate anonymised veterinary clinical data from the VetCompass Programme to explore whether demographic information on dogs such as breed, bodyweight and body shape could be useful to predict osteosarcoma. VetCompass shares anonymised veterinary clinical records for welfare-focused research. This study explored the records of 905,552 dogs under veterinary care in 2016 to identify all cases of osteosarcoma. Advanced statistical methods were used to evaluate links between demographic factors and the risk of osteosarcoma. From the overall study population of 905,552 dogs, there were 331 osteosarcoma cases identified to show a one-year period prevalence of 0.037%. The breeds with the highest frequency of osteosarcoma were the Scottish Deerhound (3.28%), Leonberger (1.48), Great Dane (0.87%), Rottweiler (0.84%) and Greyhound (0.62%). Eleven breeds showed increased risk of osteosarcoma compared with crossbred dogs. Breeds with the highest risk included Scottish Deerhound (× 118.40 times risk), Leonberger (× 55.79), Great Dane (× 34.24) and Rottweiler (× 26.67). Aging was progressively and strongly associated with increasing risk of osteosarcoma. Dogs weighing heavier that the average for their breed had 1.65 times the risk than animals weighing below the breed average. Insured dogs had 1.71 times the risk of being diagnosed with osteosarcoma compared with uninsured dogs which may indicate higher levels of healthcare given to insured dogs compared to uninsured dogs. Chondrodystrophic (short-legged) breeds had 0.10 times the risk of osteosarcoma compared with non-chondrodystrophic breeds. Compared with breeds with mesocephalic (average length) skull conformation, breeds with dolichocephalic (long) skull conformation (× 2.72) had increased odds of osteosarcoma while breeds with brachycephalic (short) skull conformation showed reduced risk (× 0.50). This study cements the concept that breed, bodyweight and longer leg or longer skull length all predispose to osteosarcoma in dogs. With this awareness, veterinarians can update their clinical suspicion and judgement, breeders can select towards lower-risk animals, and researchers can define more useful study populations for better research.

**Supplementary Information:**

The online version contains supplementary material available at 10.1186/s40575-023-00131-2.

## Background

Osteosarcoma describes a malignant bone tumour that is reported in several species but most commonly in dogs and humans [1–4]. Affected dogs often present with lameness with a bony or soft tissue swelling that is associated with severe, sometimes waxing and waning, discomfort, while pathological fractures are a common complication of osteosarcoma [2, 5]. Therefore, osteosarcoma is considered a significant welfare concern for affected dogs due to ongoing pain despite analgesia administration [2, 5]. Radiographically, osteosarcomas appear as aggressive bone lesions (cortical destruction, punctate radiolucencies, irregular periosteal reaction, new bone formation and long transition zone). Biopsy is recommended for diagnostic confirmation [4, 6, 7]. Osteosarcoma generally occurs at specific anatomical locations, most often at the metaphyseal regions of the appendicular skeleton, especially the proximal humerus, distal femur, and proximal or distal tibia. This suggests that some as-yet undefined aspects of the biology of physis closure and metaphyseal bone growth, or the mechanics of load on physeal regions may be involved in osteosarcoma pathogenesis [2, 4, 8]. Appendicular osteosarcomas usually follow an aggressive clinical course, with 90% of canine patients presenting with microscopic or gross pulmonary metastases at the time of diagnosis [2, 4, 9]. Axial skeleton osteosarcomas are commonly less clinically aggressive than appendicular lesions, whereas those of the extraskeletal tissues (e.g. mammary gland, subcutaneous tissue, gastro-intestinal tract) are rarer but more aggressive [10, 11]. Pertinent differential diagnoses for alternative aggressive bone lesions include bacterial or fungal osteomyelitis, chondrosarcoma, fibrosarcoma or haemangiosarcoma. A final diagnosis of osteosarcoma can be confirmed via histopathology after surgical resection [12, 13].

There are many studies investigating the parallels between canine and human osteosarcoma, encompassing patient characteristics, clinical course and tumour biology. The similarities of these bone neoplasia between the species support the value of studies on canine osteosarcoma in informing human translational medicine. Although osteosarcoma in humans is rare (affecting 3 in 1 million UK individuals each year), it follows a similarly aggressive clinical course to the dog. Patients generally succumb rapidly to metastatic disease, reflected in human median survival rates of 60% at 5-years with standard of care therapy [2, 4, 11, 14–17]. Both humans and dogs exhibit peaks of osteosarcoma incidence in adolescence relative to their species’ development (at 2-years in a recent canine study and 15–19 years in humans in the UK), and it is hypothesized that this juvenile patient population inherit increased osteosarcoma risk via genetic variants [18–20]. However, a second peak in cases of osteosarcoma in middle aged to older dogs and people is attributed to typical age-associated cancer risk, as with many other cancers [2, 14, 16–18, 21–23]. A number of rare, high-effect variants that drive osteosarcoma have been identified in human adolescents. For example, children carrying the TP53 mutation in Li Fraumeni syndrome, and those with the RB1 mutation in hereditary retinoblastoma are at very high lifetime risk of many cancers, including osteosarcoma (> 90% all-cancer risk in females with Li Fraumeni). In dogs, osteosarcoma is likely driven by a combination of higher frequency, low-effect variants, which are inherited in combination owing to selective breeding and breed-associated genetic architecture. Such combinations of commonly carried low-effect alleles may contribute to osteosarcoma risk but be challenging to detect in human populations owing to the small effect size of each individual variant [21, 24]. Thus, the dog acts as an important model for osteosarcoma genomics. The largest canine genome-wide association study (GWAS) to-date considered only three breeds, identifying the CDKN2A locus as site for important osteosarcoma risk variants [24–28]. Environmental factors are also required alongside genetics to create the perfect environment for osteosarcoma development [22, 29].

Earlier epidemiological studies into canine osteosarcoma, including by our group, may have been subject to selection bias because the case sampling was limited to histologically confirmed diagnoses, referral populations or to histopathology laboratory databases [2, 17, 19, 21–23, 30–37]. In the current study, we build and extend on such previous work by interrogating anonymised veterinary clinical data from the VetCompass Programme [38] to explore associations between risk factors, especially breed, bodyweight and body conformation, with osteosarcoma in dogs [18, 19, 21, 23, 31–33]. Using a database of clinical information associated with osteosarcoma biopsies, our group previously reported that some common conformational traits in dogs such as chondrodystrophy and brachycephaly were associated with protection from osteosarcoma, implying that the genetic control of bone growth may play a role in osteosarcoma risk [21]. Therefore, a particular aim of the current work was to validate that finding in a different and larger patient population. In using a larger database originating from primary-care veterinary records, we identified risk factors associated with osteosarcoma, and then employed data triangulation to determine if the current findings persisted across several studies [39]. In doing so, we were able to provide greater confidence in the biological relevance of such findings to the phenomenon of osteosarcoma. The results we present here could assist veterinary practitioners to prepare owners with improved awareness of higher risk of osteosarcoma in predisposed breeds and therefore promote earlier presentation of cases that could lead to better clinical and welfare outcomes [40]. In addition, stronger evidence on the links between extreme conformations such as giantism with heightened osteosarcoma risk can support ongoing efforts by welfare scientists and breeders to breed away from extreme conformations that reduce innate health [41, 42]. These results also provide a platform for better fundamental bioscience [43].

Based on previous work, the current study hypothesized that dogs with larger bodyweight, dolichocephalic conformation and non-chondrodystrophic breed status have greater odds of osteosarcoma than dogs of lower bodyweight, mesocephalic/brachycephalic conformation and chrondrodystrophic breed status respectively. We hypothesized that breed overall would be significantly associated with osteosarcoma, as previously reported [21, 23, 33].

## Results

### Prevalence

**Fig. 1:**
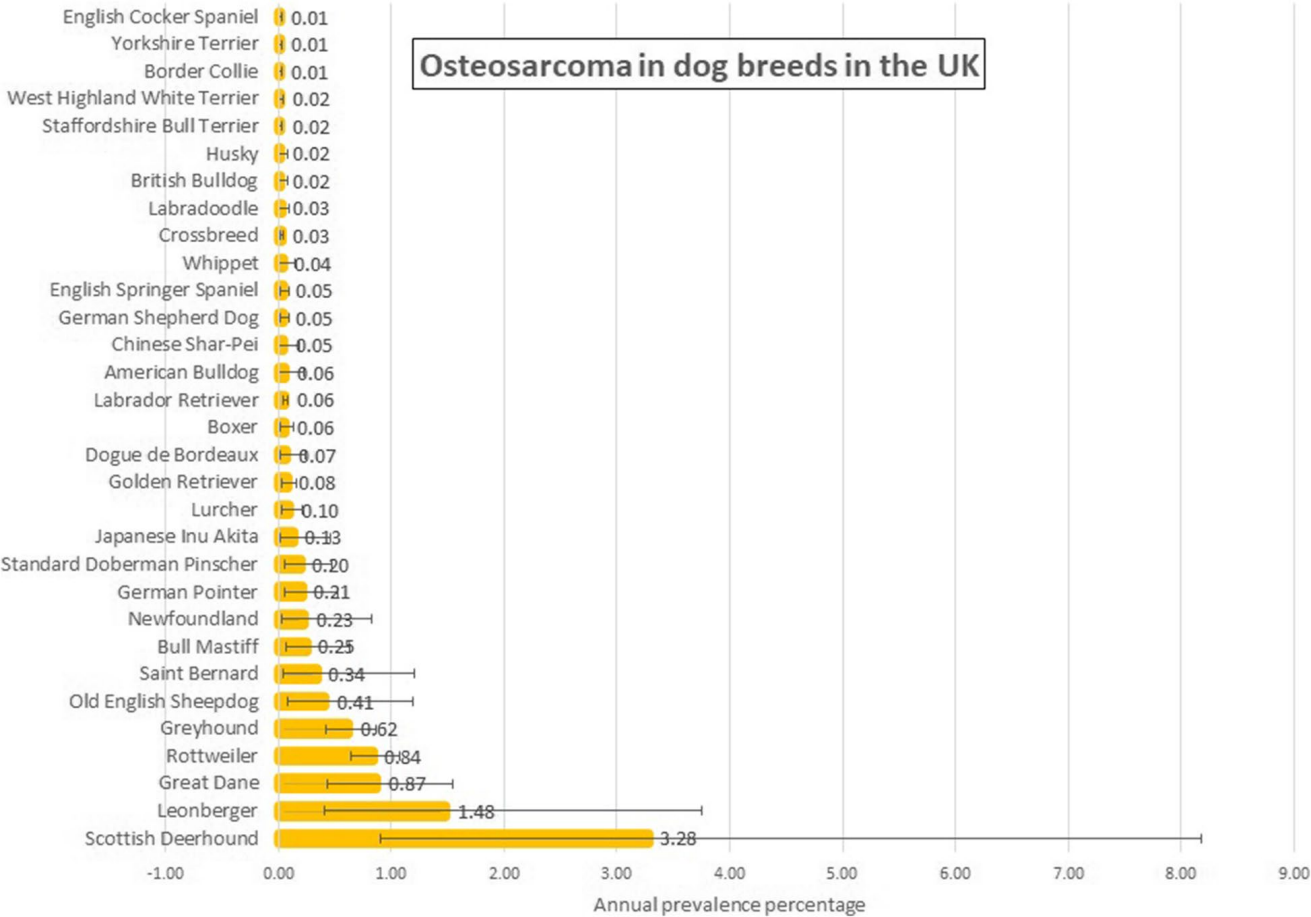
One-year (2016) period prevalence (percentage) of osteosarcoma in dog breeds under primary veterinary care in the VetCompass Programme in the UK. The horizontal bars represent 95% confidence intervals

From an overall study population of 905,552 dogs under veterinary care in 2016 at 887 veterinary clinics, 331 osteosarcoma cases were confirmed during 2016, yielding a one-year period prevalence for osteosarcoma in dogs overall of 0.037% (95% CI: 0.033–0.041). Of these cases, 20 were first diagnosed prior to 2016 and 311 were first diagnosed during 2016, yielding an annual incidence risk of 0.034 (95% CI: 0.031–0.038). Among breeds with at least 2 cases of osteosarcoma diagnosed during 2016, breeds with the highest osteosarcoma annual prevalence were the Scottish Deerhound (3.28%, 95% CI 0.90–8.18), Leonberger (1.48%, 95% CI 0.41- 3.75), Great Dane (0.87%, 95% CI 0.43- 1.55), Rottweiler (0.84%, 95% CI 0.64–1.07) and Greyhound (0.62%, 95% CI 0.43–0.87) (Fig. 1). Details of all breeds with at least one case of osteosarcoma are presented in Additional file 1: Supplementary A. These results show the breeds with the highest proportions of dogs diagnosed with osteosarcoma.

**Fig. 2:**
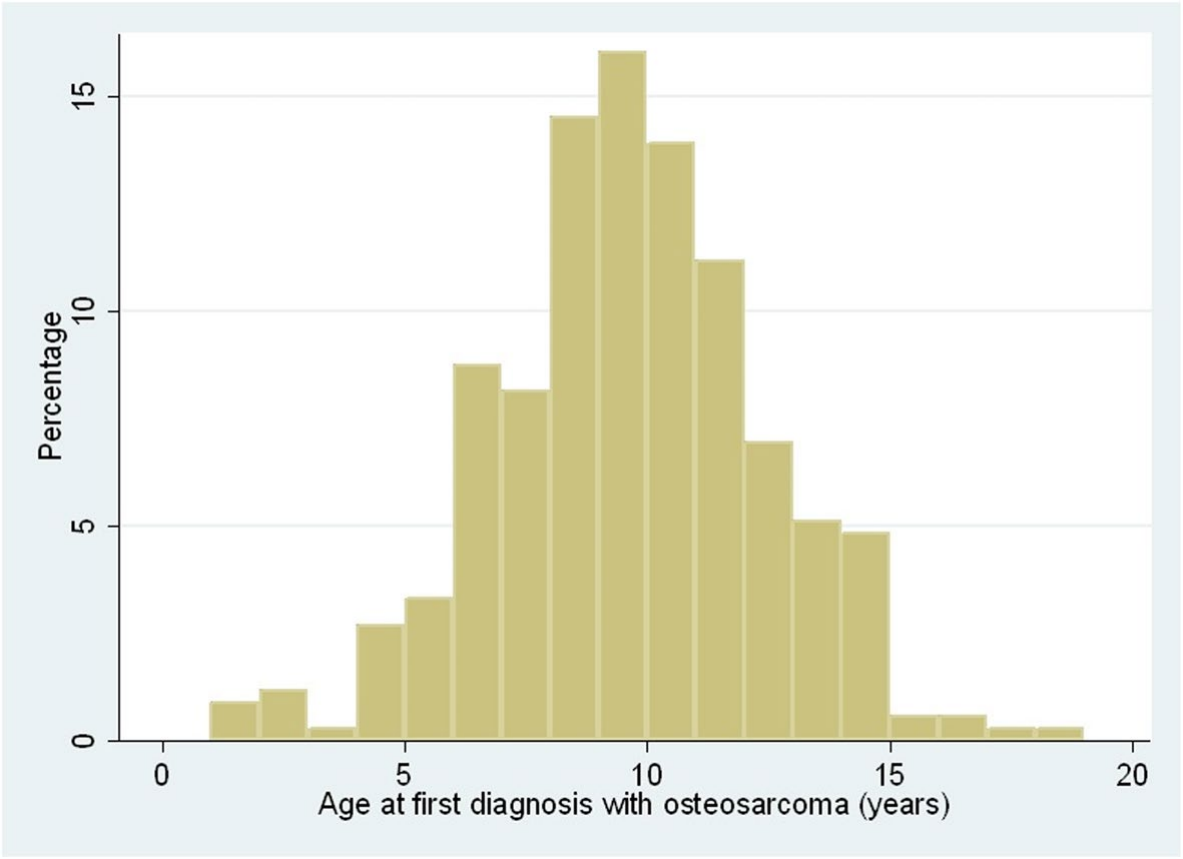
Age (years) at first diagnosis with osteosarcoma in dogs under primary veterinary care in the VetCompass Programme in the UK

Of the osteosarcoma cases with data available for that variable, 261 (78.85%) were purebred, 167 (50.45%) were female and 215 (64.95%) were neutered. Dogs with osteosarcoma had a median adult bodyweight of 33.04 kg (IQR: 25.50–42.93, range 7.40–75.87) and median age at diagnosis was 9.64 years (IQR: 7.97–11.41, range 1.20–18.01) (Fig. 2). The most common breeds among the osteosarcoma cases were Rottweiler (n = 61, 18.43% of all cases), Labrador Retriever (38, 11.48%), Greyhound (34, 10.27%), German Shepherd Dog (11, 3.32%), Great Dane (11, 3.32%) and Staffordshire Bull Terrier (11, 3.32%) 68.88, along with Crossbreed (62, 18.73%) (Table 1). These results show the breeds that comprise the greatest component of the overall caseload of osteosarcoma cases under primary veterinary care.

**Table 1:** Descriptive and univariable logistic regression results for breed as a risk factor for osteosarcoma during 2016 in dogs under primary veterinary care in the VetCompass Programme in the UK. Column percentages shown in brackets. ^a^CI confidence interval

Breed	Case No. (%)	Non-case No. (%)	Odds ratio	95% CI^a^	Category *P*-value	Variable *P*-value
Crossbreed	62 (18.73)	194,429 (21.48)	Base			< 0.001
Scottish Deerhound	4 (1.21)	118 (0.01)	106.30	38.06–296.90	< 0.001	
Leonberger	4 (1.21)	266 (0.03)	47.16	17.03–130.54	< 0.001	
Great Dane	11 (3.32)	1258 (0.14)	27.42	14.41–52.19	< 0.001	
Rottweiler	61 (18.43)	7223 (0.80)	26.48	18.58–37.74	< 0.001	
Greyhound	34 (10.27)	5422 (0.60)	19.66	12.93–29.90	< 0.001	
Old English Sheepdog	3 (0.91)	734 (0.08)	12.82	4.01–40.92	< 0.001	
Bull Mastiff	4 (1.21)	1620 (0.18)	7.74	2.81–21.31	< 0.001	
German Pointer	4 (1.21)	1921 (0.21)	6.53	2.37–17.97	< 0.001	
Standard Doberman Pinscher	5 (1.51)	2456 (0.27)	6.38	2.56–15.89	< 0.001	
Lurcher	6 (1.81)	6016 (0.66)	3.13	1.35–7.23	0.008	
Golden Retriever	8 (2.42)	9785 (1.08)	2.56	1.23–5.36	0.012	
Labrador Retriever	38 (11.48)	59,925 (6.62)	1.99	1.33–2.98	0.001	
Boxer	6 (1.81)	9438 (1.04)	1.99	0.86–4.61	0.107	
German Shepherd Dog	11 (3.32)	21,360 (2.36)	1.61	0.85–3.07	0.143	
English Springer Spaniel	10 (3.02)	20,198 (2.23)	1.55	0.80–3.03	0.197	
Labradoodle	2 (0.60)	7483 (0.83)	0.84	0.20–3.43	0.806	
English Bulldog	2 (0.60)	8407 (0.93)	0.75	0.18–3.05	0.683	
Husky	2 (0.60)	8563 (0.95)	0.73	0.18–2.99	0.665	
Breed type—Others	30 (9.06)	136,328 (15.06)	0.69	0.45–1.07	0.095	
Staffordshire Bull Terrier	11 (3.32)	53,045 (5.86)	0.65	0.34–1.23	0.188	
West Highland White Terrier	3 (0.91)	18,875 (2.09)	0.50	0.16–1.59	0.239	
Beagle	1 (0.30)	8069 (0.89)	0.39	0.05–2.80	0.348	
Border Collie	2 (0.60)	24,388 (2.69)	0.26	0.06–1.05	0.059	
Yorkshire Terrier	2 (0.60)	28,178 (3.11)	0.22	0.05–0.91	0.037	
English Cocker Spaniel	2 (0.60)	33,075 (3.65)	0.19	0.05–0.78	0.021	
Pug	1 (0.30)	16,213 (1.79)	0.19	0.03–1.39	0.103	
Shih-tzu	1 (0.30)	32,909 (3.64)	0.10	0.01–0.69	0.020	
Jack Russell Terrier	1 (0.30)	48,569 (5.37)	0.06	0.01–0.47	0.007	
Bichon Frise	0 (0.00)	13,269 (1.47)	~			
Border Terrier	0 (0.00)	9651 (1.07)	~			
Cavalier King Charles Spaniel	0 (0.00)	17,258 (1.91)	~			
Chihuahua	0 (0.00)	36,794 (4.06)	~			
Cockapoo	0 (0.00)	18,404 (2.03)	~			
French Bulldog	0 (0.00)	16,397 (1.81)	~			
Lhasa Apso	0 (0.00)	12,549 (1.39)	~			
Miniature Schnauzer	0 (0.00)	8397 (0.93)	~			
Pomeranian	0 (0.00)	6221 (0.69)	~			

Of the dogs that were not osteosarcoma cases with data available on the variable, 654,647 (72.58%) were purebred and 431,540 (47.90%) were female, 407,750 (45.26%) were neutered. The median adult bodyweight for non-cases was 13.95 kg (IQR: 8.19–25.00, range 0.72–97.20) and the median age was 4.44 years (IQR: 1.87–8.08, range 0.00–20.97). The most common breeds among the non-case dogs were Labrador Retriever (59,925, 6.62% of non-cases), Staffordshire Bull Terrier (52,045, 5.86%), Jack Russell Terrier (48,596, 5.37%), Chihuahua (36,794, 4.06%) and English Cocker Spaniel (33,075, 3.65%), along with Crossbred (n = 194,429, 21.48%) (Table 1).

### Risk factors

All study variables were liberally associated with osteosarcoma in univariable logistic regression modelling and were evaluated using multivariable logistic regression modelling (Tables 1, 2 & 3). The final breed-focused multivariable model retained four risk factors: *breed, bodyweight relative to breed mean, age, and insurance* (Table 4) with other risk factors dropped because they were not statistically significantly associated with the osteosarcoma outcome or were not identified as showing a confounder/interaction effect. *Sex-neuter* and *vet group* were not associated with the odds of osteosarcoma and therefore were not retained in the final model. No biologically significant interactions were identified. The final model showed acceptable model-fit (Hosmer–Lemeshow test statistic: *P* = 0.364) and good discrimination (area under the ROC curve: 0.909).

**Table 2:** Descriptive and univariable logistic regression results for breed-derived risk factors for osteosarcoma during 2016 in dogs under primary veterinary care in the VetCompass Programme in the UK. Column percentages shown in brackets. ^a^CI confidence interval

Variable	Category	Case No. (%)	Non-case No. (%)	Odds ratio	95% CI^a^	Category *P*-value	Variable *P*-value
Breed purity	Crossbred	62 (18.73)	194,429 (21.56)	Base			0.003
	Designer	8 (2.42)	52,863 (5.86)	0.47	0.23–0.99	0.047	
	Purebred	261 (78.85)	654,647 (72.58)	1.25	0.95–1.65	0.114	
Kennel Club Recognised Breed	Not recognised	75 (22.66)	262,287 (29.08)	Base			0.008
	Recognised	256 (77.34)	639,652 (70.92)	1.40	1.08–1.81	0.010	
Kennel Club Breed Group	Not Kennel Club recognised breed	75 (22.66)	262,287 (29.08)	Base			< 0.001
	Terrier	15 (4.53)	145,912 (16.18)	0.36	0.21–0.63	< 0.001	
	Gundog	66 (19.94)	135,607 (15.04)	1.70	1.22–2.37	0.002	
	Working	102 (30.82)	39,114 (4.34)	9.12	6.77–12.29	< 0.001	
	Pastoral	19 (5.74)	52,964 (5.87)	1.25	0.76–2.08	0.377	
	Utility	8 (2.42)	102,655 (11.38)	0.27	0.13–0.56	< 0.001	
	Hound	42 (12.69)	31,376 (3.48)	4.68	3.21–6.83	< 0.001	
	Toy	4 (1.21)	132,024 (14.64)	0.11	0.04–0.29	< 0.001	
Chondrodystrophy	Non chondrodystrophic	238 (71.90)	309,279 (34.17)	Base			< 0.001
	Chondrodystrophic	23 (6.95)	345,327 (38.15)	0.09	0.06–0.13	< 0.001	
	Uncategorised	70 (21.15)	250,605 (27.68)	0.36	0.27–0.47	< 0.001	
Skull conformation	Mesocephalic	169 (51.06)	417,452 (46.12)	Base			< 0.001
	Brachycephalic	20 (6.04)	167,413 (18.49)	0.30	0.19–0.47	< 0.001	
	Dolichocephalic	72 (21.75)	69,782 (7.71)	2.55	1.93–3.36	< 0.001	
	Uncategorised	70 (21.15)	250,564 (27.68)	0.69	0.52–0.91	0.009	
Haircoat length	Medium	44 (13.29)	191,904 (21.20)	Base			< 0.001
	Short	210 (63.44)	339,558 (37.51)	2.70	1.95–3.73	< 0.001	
	Long	7 (2.11)	92.018 (10.17)	0.33	0.15–0.74	0.007	
	Uncategorised	70 (21.15)	281,731 (31.12)	1.08	0.74–1.58	0.676

**Table 3:** Descriptive and univariable logistic regression results for non-breed-related demographic risk factors evaluated for osteosarcoma during 2016 in dogs under primary veterinary care in the VetCompass Programme in the UK. Column percentages shown in brackets. ^a^CI confidence interval

Variable	Category	Case No. (%)	Non-case No. (%)	Odds ratio	95% CI^a^	Category *P*-value	Variable *P*-value
Adult (> 18 months) bodyweight (kg)	< 10.0	4 (1.21)	213,321 (23.57)	0.13	0.05–0.38	< 0.001	
	10.0—< 20.0	24 (7.25)	167,774 (18.53)	Base			< 0.001
	20.0—< 30.0	64 (19.34)	117,620 (12.99)	3.80	2.38–6.08	< 0.001	
	30.0—< 40.0	78 (23.56)	69,856 (7.72)	7.81	4.94–12.33	< 0.001	
	40.0—< 50.0	48 (14.50)	19,813 (2.19)	16.94	10.37–27.65	< 0.001	
	50.0—< 60.0	25 (7.55)	4,452 (0.49)	39.26	22.40–68.78	< 0.001	
	≥ 60.0	8 (2.42)	1,913 (0.21)	29.23	13.12–65.15	< 0.001	
	Uncategorised	80 (24.17)	310,462 (34.30)	1.80	1.14–2.84	0.011	
Bodyweight relative to breed mean	Lower	91 (27.49)	317,257 (35.05)	Base			< 0.001
	Equal/Higher	160 (48.34)	275,353 (30.42)	2.03	1.57–2.62	< 0.001	
	Uncategorised	80 (24.17)	312,601 (34.53)	0.89	0.66–1.20	0.457	
Age (years)	< 4.0	8 (2.42)	412,142 (45.53)	0.14	0.06–0.31	< 0.001	
	4.0—< 6.0	20 (6.04)	139,739 (15.44)	Base			< 0.001
	6.0—< 8.0	56 (16.92)	113,515 (12.54)	3.45	2.07–5.74	< 0.001	
	8.0—< 10.0	101 (30.51)	90,840 (10.04)	7.77	4.81–12.55	< 0.001	
	10.0—< 12.0	83 (25.08)	66,450 (7.34)	8.73	5.36–14.22	< 0.001	
	12.0—< 14.0	40 (12.08)	41,950 (4.63)	6.66	3.89–11.40	< 0.001	
	≥ 14.0	22 (6.65)	28,152 (3.11)	5.46	2.98–10.01	< 0.001	
	Uncategorised	1 (0.30)	12,423 (1.37)	0.56	0.08–4.19	0.574	
Sex-neuter	Female entire	54 (16.31)	233,772 (25.83)	Base			< 0.001
	Female neutered	113 (34.14)	197,768 (21.85)	2.47	1.79–3.42	< 0.001	
	Male entire	62 (18.73)	259,460 (28.66)	1.03	0.72–1.49	0.856	
	Male neutered	102 (30.82)	209,982 (23.20)	2.10	1.51–2.92	< 0.001	
	Uncategorised	0 (0.00)	4,229 (0.47)	~			
Insurance	Non-insured	252 (76.13)	787,723 (87.02)	Base			< 0.001
	Insured	79 (23.87)	117,488 (12.98)	2.10	1.63–2.71	< 0.001	
Vet group	A	2 (0.60)	3,913 (0.43)	1.57	0.39–6.35		
	B	81 (24.47)	297,571 (32.87)	0.84	0.63–1.10		
	C	24 (7.25)	40,576 (4.48)	1.82	1.18–2.81		
	D	92 (27.79)	157,387 (17.39)	1.80	1.38–2.35		
	E	132 (39.88)	405,764 (44.83)	Base			< 0.001

**Table 4:** Multivariable logistic regression results that *includes breed* for demographic risk factors evaluated for osteosarcoma during 2016 in dogs under primary veterinary care in the VetCompass™ Programme in the UK. Column percentages shown in brackets. ^a^CI confidence interval. ~ count data too low to calculate

Variable	Category	Odds ratio	95% CI^a^	Category *P*-value	Variable *P*-value
Breed	Crossbreed	Base			** < 0.001**
	Scottish Deerhound	118.40	41.12–340.95	** < 0.001**	
	Leonberger	55.79	19.68–158.15	** < 0.001**	
	Great Dane	34.24	17.81–65.83	** < 0.001**	
	Rottweiler	26.67	18.57–38.29	** < 0.001**	
	Greyhound	11.93	7.82–18.21	** < 0.001**	
	Old English Sheepdog	9.76	3.05–31.29	** < 0.001**	
	Bull Mastiff	9.21	3.33–25.48	** < 0.001**	
	Standard Doberman Pinscher	6.23	2.49–15.58	** < 0.001**	
	German Pointer	5.05	1.83–13.94	**0.002**	
	Lurcher	2.74	1.18–6.34	**0.019**	
	Golden Retriever	1.81	0.87–3.79	0.115	
	Boxer	1.58	0.68–3.66	0.288	
	Labrador Retriever	1.57	1.05–2.36	**0.029**	
	German Shepherd Dog	1.53	0.81–2.92	0.193	
	English Bulldog	1.51	0.37–6.21	0.565	
	English Springer Spaniel	1.21	0.62–2.37	0.571	
	Labradoodle	1.15	0.28–4.71	0.848	
	Husky	1.09	0.27–4.47	0.902	
	Breed type—Others	0.76	0.49–1.17	0.213	
	Staffordshire Bull Terrier	0.53	0.28–1.01	0.055	
	Beagle	0.52	0.07–3.72	0.511	
	Pug	0.46	0.06–3.32	0.440	
	West Highland White Terrier	0.29	0.09–0.92	**0.036**	
	Border Collie	0.21	0.05–0.84	**0.028**	
	Yorkshire Terrier	0.19	0.05–0.77	**0.020**	
	English Cocker Spaniel	0.18	0.04–0.74	**0.017**	
	Shih-tzu	0.12	0.02–0.87	**0.035**	
	Jack Russell Terrier	0.05	0.01–0.34	**0.002**	
	Bichon Frise	~			
	Border Terrier	~			
	Cavalier King Charles Spaniel	~			
	Chihuahua	~			
	Cockapoo	~			
	French Bulldog	~			
	Lhasa Apso	~			
	Miniature Schnauzer	~			
	Pomeranian	~			
Bodyweight relative to breed mean	Lower	Base			** < 0.001**
	Equal/Higher	1.65	1.27–2.13	** < 0.001**	
	Uncategorised	1.43	1.05–1.94	**0.021**	
Age (years)	< 4.0	0.15	0.07–0.35	** < 0.001**	
	4.0—< 6.0	Base			** < 0.001**
	6.0—< 8.0	3.25	1.95–5.42	** < 0.001**	
	8.0—< 10.0	6.80	4.20–11.00	** < 0.001**	
	10.0—< 12.0	7.89	4.83–12.88	** < 0.001**	
	12.0—< 14.0	7.22	4.20–12.40	** < 0.001**	
	≥ 14.0	8.01	4.33–14.8	** < 0.001**	
	Uncategorised	0.53	0.07–3.98	0.535	
Insurance	Non-insured	Base			** < 0.001**
	Insured	1.71	1.33–2.22	** < 0.001**	

After accounting for the effects of the other variables evaluated, 11 breeds showed increased odds of osteosarcoma compared with crossbred dogs. Breeds with the highest odds included Scottish Deerhound (OR 118.40, 95% CI 41.12–340.95), Leonberger (OR 55.79, 95% CI 19.68–158.15), Great Dane (OR 34.24, 95% CI 17.81–65.83) and Rottweiler (OR 26.67, 95% CI 18.57–38.29). Six breeds showed reduced odds of osteosarcoma compared with crossbreds, while zero osteosarcoma cases were recorded in a further nine breeds. Aging was progressively and strongly associated with increasing odds of osteosarcoma; dogs aged 10.0 to < 12.0 years had 7.89 times the odds (95% CI 4.83–12.88) compared with dogs aged 4.0 to < 6.0 years. Dogs weighing at or above the mean for their breed had 1.65 times the odds (95% CI 1.27–2.13) than animals weighing below the breed mean. Insured dogs had 1.71 (95% CI 1.33–2.22) times the odds of being diagnosed with osteosarcoma compared with uninsured dogs (Table 4).

As described in the methods, breed-derived variables were introduced individually to replace *breed* in the final breed-focused model. Purebred status or being a Kennel Club recognised breed were not associated with the odds of osteosarcoma. Compared to breeds that were not recognised by the Kennel Club, two Kennel Club breed groups showed higher odds of osteosarcoma (Working and Hound) while three showed reduced odds (Utility, Toy and Terrier). Chondrodystrophic breeds had 0.10 times the odds (95% CI 0.06–0.15) of osteosarcoma compared with non-chondrodystrophic breeds. Compared with breeds with mesocephalic skull conformation, breeds with dolichocephalic skull conformation (OR 2.72, 95% CI 2.06–3.58) had increased odds of osteosarcoma while breeds with brachycephalic skull conformation showed reduced odds (OR 0.50, 95% CI 0.32–0.80). Compared with breeds with medium length coats, breeds with short hair showed increased odds of osteosarcoma (OR 3.08, 95% CI 2.22–4.26) while breeds with long hair showed reduced odds (OR 0.41, 95% CI 0.19–0.92). Increasing adult bodyweight was associated with increasing odds of osteosarcoma (Table 5).

**Table 5:** Multivariable logistic regression results for variables that replaced *breed* in risk factor analysis for osteosarcoma during 2016 in dogs under primary veterinary care in the VetCompass™ Programme in the UK. Column percentages shown in brackets. ^a^CI confidence interval. (note: Adult bodyweight replaced both breed and relative bodyweight)

Variable	Category	Odds ratio	95% CI^a^	Category *P*-value	Variable *P*-value
Breed purity	Crossbred	Base			0.613
	Designer	1.10	0.53–2.32	0.794	
	Purebred	1.15	0.87–1.52	0.329	
Kennel Club Recognised Breed	Not recognised	Base			0.373
	Recognised	1.12	0.87–1.45	0.378	
Kennel Club Breed Group	Not Kennel Club recognised breed	Base			** < 0.001**
	Working	8.70	6.43–11.79	** < 0.001**	
	Hound	3.91	2.67–5.72	** < 0.001**	
	Gundog	1.21	0.87–1.69	0.264	
	Pastoral	0.92	0.55–1.52	0.736	
	Utility	0.31	0.15–0.64	**0.002**	
	Terrier	0.23	0.13–0.40	** < 0.001**	
	Toy	0.11	0.04–0.31	** < 0.001**	
Chondrodystrophy	Non chondrodystrophic	Base			** < 0.001**
	Chondrodystrophic	0.10	0.06–0.15	** < 0.001**	
	Uncategorised	0.48	0.37–0.63	** < 0.001**	
Skull conformation	Mesocephalic	Base			** < 0.001**
	Brachycephalic	0.50	0.32–0.80	**0.004**	
	Dolichocephalic	2.72	2.06–3.58	** < 0.001**	
	Uncategorised	0.96	0.73–1.27	0.791	
Haircoat length	Medium	Base			** < 0.001**
	Short	3.08	2.22–4.26	** < 0.001**	
	Long	0.41	0.19–0.92	**0.031**	
	Uncategorised	1.60	1.09–2.33	**0.015**	
Adult (> 18 months) bodyweight (kg)	< 10.0	0.15	0.05–0.43	** < 0.001**	
	10.0—< 20.0	Base			** < 0.001**
	20.0—< 30.0	3.39	2.12–5.42	** < 0.001**	
	30.0—< 40.0	6.68	4.22–10.56	** < 0.001**	
	40.0—< 50.0	14.54	8.89–23.78	** < 0.001**	
	50.0—< 60.0	43.12	24.52–75.82	** < 0.001**	
	≥ 60.0	36.62	16.35–82.01	** < 0.001**	
	Uncategorised	3.51	2.22–5.55	** < 0.001**	

## Discussion

In the current study, we investigated a database of primary care veterinary practice records from VetCompass to identify dogs diagnosed with osteosarcoma (cases) and those without (non-cases). These data were used to determine risk factors associated with osteosarcoma in dogs, as well as the frequency of osteosarcoma amongst the entire population and within breeds. Strengths of the study included its very large population size, the high degree of statistical certainty associated with the results (as shown by p-values and confidence intervals) and the measures taken to limit sampling biases.

Firstly, including a denominator population of all dogs under care at participating veterinary practices reduced the likelihood of selection bias, which may occur within a case-only dataset or with datasets from referral care or pathology laboratories [35, 44, 45]. An example of selection bias in veterinary medicine is the phenomenon by which analysis of cases alone cannot differentiate whether breeds that feature highly in a dataset of osteosarcoma cases do so because they are truly at high risk of the cancer or because they are simply common breeds in the wider general population [46]. This is illustrated in the current analysis, because Staffordshire Bull Terriers and Labrador Retrievers feature highly in the osteosarcoma dataset, but also in dogs in the UK, meaning that they did not appear as high-risk breeds once the denominator population was utilised. Secondly, in the current analysis we aimed to reduce collider bias by avoiding the need for a confirmed histologic diagnosis for a case to be classified as osteosarcoma, as discussed below. Although this generates a degree of diagnostic uncertainty (e.g. some dogs with osteomyelitis or another aggressive bone neoplasm may have been misclassified as osteosarcoma cases in the study), we considered it likely that most diagnoses of osteosarcoma made using radiography would have been correct, given the relative rarity of monostotic lesions associated with bacterial and fungal osteomyelitis and other bone neoplasms in the UK [47]. The concept of collider bias has been reviewed previously [34, 35]. Briefly, collider bias occurs when two conditions are required for enrolment in the study, leading to the identification of a falsely inflated degree of association between those conditions. For example, if socioeconomic status determines that all histologically confirmed cases of osteosarcoma also occur in neutered dogs, we may falsely assume an association between neutering and osteosarcoma [34, 35]. Fortunately, although no single epidemiologic study is fully immune from bias or confounding, multiple data sources are now available for osteosarcoma in dogs, each utilising different patient populations [21, 23, 32, 48]. Therefore, when considering the biological relevance of findings, triangulation across several study designs can compare the current results to previous studies, with an acceptance that findings that persist across analyses are more likely to be truly biologically valid. In the current study, findings were triangulated with a previous large analysis of clinical data associated with histologically confirmed osteosarcomas, further increasing our confidence that the findings presented are relevant [45].

The four breeds at greatest risk of osteosarcoma in the current study were the Scottish Deerhound (OR 118, 95% CI 41.12 – 340.95), Leonberger (OR 55.79, 95% CI 19.68 – 158.15), Great Dane (OR 34.24, 95% CI 17.81 – 65.83) and Rottweiler (OR 26.67, 95% CI 18.57 – 38.29). The odds ratios for these breeds were very high, with lower confidence limits suggesting that the most at-risk breeds possess odds of at least 18 to 40 times higher than crossbreeds, even accounting for statistical uncertainty and for other confounding variables. Other highly at-risk breeds included the Greyhound, Old English Sheepdog, Bull Mastiff, Doberman, German Pointer and Lurcher. It should be noted that previous work in VetCompass has reported that greyhounds under primary veterinary care are typically older (7.6 years) than other dogs (4.4 years) and therefore this greyhound cohort is likely to reflect a large proportion of retired ex-racing dogs rather than younger racing dogs [49, 50], although some greyhounds are bred specifically as companion or show animals [51]. These overall breed findings are largely consistent with clinical and epidemiological observations to-date [21, 23, 32, 48]. Previous studies in the UK and the US have also attributed a high osteosarcoma risk to the Golden Retriever, which was the twelfth most at-risk breed in the current study, although this effect did not meet the study-wide p-value threshold. Notably, the Rhodesian Ridgeback was also absent from the current study, where our group previously found this breed to be highly at-risk [45]. As well as representing true differences across the study populations, failure to replicate consistent findings for these two breeds in the current study may result from limited breed-based study power or may reflect other biases. The gap between the very high odds ratios reported for breeds such as the Scottish Deerhound, and more moderate odds ratios present in breeds such as the Golden Retriever, may also point to different modes of inheritance. It has been proposed that Scottish Deerhounds may carry mendelian level inheritance of high-effect osteosarcoma risk variants, whereas other breeds may require complex inheritance of polygenic risk to be at risk [24, 52, 53]. Overall, the current study supports the idea that multiple methodologies (familial inheritance studies, GWAS, whole genome sequencing) should be used across breeds to generate a broader picture of how dogs inherit their osteosarcoma risk or protection.

Identifying those breeds at reduced risk of osteosarcoma (protected breeds) may be just as valuable as identifying those at increased risk, because protected breeds could be used to identify factors which reduce the risk of disease. In veterinary medicine, studies are increasingly better equipped to identify protected breeds thanks to the availability of Big Data study populations such as VetCompass [21, 38, 54, 55]. Many of the protected breeds identified in the current study were also triangulated in previous analyses, including the Border Collie, Cocker Spaniel, West Highland White Terrier and Jack Russell Terrier [21]. The Bichon Frise, Cavalier Kind Charles Spaniel and French Bulldog had zero osteosarcoma cases in the current study and were the most protected in previous work, suggesting a very high degree of heritable protection [21]. The Yorkshire Terrier, Shih-Tzu, Chihuahua, Cockapoo, Lhasa Apso, Miniature Schnauzer and Pomeranian may have been missed in previous analyses due to sampling bias or small sample size in the earlier studies and therefore the potential for heritable protection against osteosarcoma in these breeds remains important, despite the fact that the current analysis is the first to report it. Notably, amongst protected breeds, small body size, short leg length, carrying chondrodystrophy genes and a brachycephalic conformation are prevalent, in keeping with the associations identified between such conformational traits and osteosarcoma risk or protection in the current study and previous analyses [21, 23].

In the current study, we considered body mass, brachycephalic status and chrondrodystrophic status as separate traits for potential association with osteosarcoma predisposition. However, we acknowledge below that there could be complex interactions between the heritability of the above traits and that of osteosarcoma risk. When considering skull shape, notably, we showed that brachycephalic dogs were protected (OR 0.50, 95% CI 0.32–0.80) and dolichocephalic dogs highly at risk (OR 2.72, 95% CI 2.06–3.58) of osteosarcoma, compared with mesocephalic dogs. This study is the second to identify associations between facial conformation and osteosarcoma risk, providing strong evidence for a role of the genes that control skull shape in the biology of osteosarcoma, or suggesting that variants associated with skull shape are inherited in linkage with those that influence osteosarcoma risk [45]. Furthermore, the odds ratios identified here were very similar to previous analyses, suggesting that risk factors related to skull shape can be interpreted with a high level of confidence. It is unclear however, whether there is a continuum of association between nose length and osteosarcoma risk, or if different sets of genes put dolichocephalic dogs at risk of osteosarcoma and mediate protection amongst brachycephalic individuals.

When considering leg length, chondrodystrophy was significantly associated with osteosarcoma protection (OR 0.10, 95% CI 0.06–0.15) compared with non-chondrodystrophic breeds, supporting the conclusions from previous analyses that shorter leg length may be protective for osteosarcoma [21]. Interestingly, in human adolescents, increased leg length at puberty is also associated with higher osteosarcoma risk [3]. Osteosarcoma lesions most commonly occur at the region of the closed physes in dogs, whereas in humans, they occur when the physes remain open [8, 13]. The link between physis biology and osteosarcoma is poorly understood and poorly reviewed in the literature. Osteosarcoma tumorigenesis may involve factors secreted by chondrocytes (the main proliferative cells of the physis) in order to recruit or regulate osteoblasts [8], as the neoplastic osteosarcoma cell is most commonly osteoblast-derived [12, 56]. It is not known whether canine physes maintain an active chondrocyte population even after closure, if closure is delayed in at risk breeds, or if an initiating event occurs during physis closure, causing lasting dysregulation of osteoblast homeostasis [8]. Nevertheless, multiple additional genetic or environmental ‘hits’ may be required to occur later in life before osteosarcoma develops, even amongst at-risk breeds. Such initiating events and subsequent hits could be related to breed largeness and leg length e.g. owing to excess growth during physeal opening, or excess mechanical loading occurring both during bone growth and after physis closure [57]. Much work is required to elucidate the genetic and environmental impacts of breed conformation on bone growth and osteosarcoma development. The dog provides an ideal model with which to interrogate the role of leg length and physis biology in human osteosarcoma, given the availability of tissue for laboratory studies from large numbers of canine patients with the disease (whereas tissue from human patients is scarce) [31].

The interplay between the different conformational traits of chondrodystrophy and brachycephaly is also interesting. As discussed elsewhere, many chondrodystrophic breeds are also brachycephalic, and genes within the bone morphogenetic protein (BMP) and fibroblast growth factor (FGF) families are drivers of both traits, making them candidates wherein risk-associated variants could be found in canine osteosarcoma [58, 59]. The effects of chondrodystrophy and brachycephaly may be additive, as exemplified by breeds such as Dogue de Bordeaux that have brachycephalic conformation but large body size and long leg length. Such breeds carried much lower risk than non-brachycephalic dogs of equivalent body size in the current analysis and previous studies, which may suggest that the effects of their brachycephaly are superimposed upon those of their other conformational traits to reduce their overall osteosarcoma risk [21]. However, the current analysis also identified high odds of osteosarcoma in the Bull Mastiff, a large brachycephalic breed, suggesting that the effect of brachycephaly may not be uniform across breeds. In wild canids with relatively long leg length, skeletal osteosarcoma is uncommonly reported, with isolated reports of the extraskeletal form present in the literature. This could suggest that the genetic and environmental characteristics associated with moderately long leg length can be achieved during prolonged evolutionary processes without increasing osteosarcoma risk, while the accelerated modern breeding of dogs for extreme conformational traits has increased the risk of certain cancers, including osteosarcoma. However, a significant cause of death in geriatric captive wild canids is neoplasia, meaning that whilst natural selection is likely to select against inheritance of risk alleles for cancers which occur in early life, cellular aging can inevitably lead to cancer in any population, even those at low heritable risk [60, 61]. The complexity of association between various conformational traits and osteosarcoma risk perhaps supports the idea of polygenic risk in many breeds, with associated environmental influences, and therefore the biology of bone in disparate breeds should be explored using further genetic and phenotypic analyses.

Adult bodyweight was significantly associated with osteosarcoma risk, in line with previous work [21, 23, 32, 48]. In the current analysis, dogs with higher-than-average bodyweight for their breed showed increased risk. Interestingly, male dogs are typically heavier than their female counterparts, and the association between male sex and osteosarcoma which has been reported previously in the literature was absent in the current analysis once bodyweight relative to breed average was taken into account [19, 23, 62]. It may be that male dogs have previously been thought to be more at risk of osteosarcoma because of their sex, when it is their relatively higher bodyweight which may actually put them at higher risk than females.

There was some evidence that neutering was associated with increased odds of osteosarcoma in the current study, however the magnitude of the odds ratios was smaller than in previous analyses [45, 63]. This supports the presence of confounding, selection or collider biases in the current or previous studies in relation to neutering results e.g. where biopsy data is used to define osteosarcoma cases, owners who can afford histology may also be better able to afford neutering, leading to an overestimation of the effect of neutering on disease risk. In addition, the neutering field in clinical records may sometimes be incorrectly completed, and the neutering information as a binary variable as used in the current study does not account for age at neutering. Therefore, overall, we consider that a prospective study design would be required to determine the effect of neutering on osteosarcoma risk, with appropriate efforts to control for the many confounders affecting the neutering variable [64].

We found a similar trend of positive association between age and osteosarcoma to previous studies [11, 21, 23]. However, in the current study, the oldest dogs (> 14 years) were most at-risk whereas previous analyses identified a peak in risk at 9–12 years with reduced risk later in life [21]. Changing canine demography with moves towards ownership of smaller dogs that live longer in combination with canine healthcare advances that promote longer lives in dogs may explain why the current study, restricted to data from 2016, identifies higher risk of osteosarcoma in older age groups compared to many earlier studies [65–67]. As well as a peak of incidence in old age, our study also supports an early incidence peak in dogs of 2–3 years old. Although the biology associated with age and osteosarcoma risk requires further research in dogs, younger osteosarcoma patients may carry relatively higher heritable risk whereas older patients may reflect a greater contribution from age-related bone cancer risk as seen in human populations [11, 21]. Unfortunately, osteosarcoma appears to be clinically aggressive in both old and young patients in both dog and human populations [2, 11, 31, 68].

Insured dogs were almost twice as likely as uninsured dogs to receive a diagnosis of osteosarcoma in the current study. Associations between insurance and diagnostic completion rates has been demonstrated previously for several other conditions such as heat-related illness and hypothyroidism using VetCompass data [69, 70]. A strong effect of insurance status on diagnostic rates holds important implications for differential care given to animals depending on their insurance status and on owners’ ability to fund veterinary care for their animals. These issues of access to good (or even adequate) veterinary care have become even more concerning as we work through the current ‘cost of living crisis’ in the UK [71] and has sparked wider veterinary discussions about the benefits of contextualised care for dogs at a population level over the gold standard approach that tends to focus on care at an individual animal level [72].This association may also be affected by collider bias, as a number of factors could be present in both insured and osteosarcoma populations that partially explain their apparent association [73]. For example, larger breed dogs that are predisposed to osteosarcoma anyhow may be more likely to be insured owing to fears about the expense associated with treating them.

The current study could influence research and clinical practice in multiple ways. Awareness of high-risk breed and body conformational traits could act as clinical alerts for veterinarians, promoting earlier detection of osteosarcoma. Conversely, lytic bone lesions identified on imaging in low risk breeds and conformations could warrant greater consideration of alternative diagnoses to osteosarcoma. Given the huge effect sizes identified in the current study in certain breeds such as the Scottish Deerhound, there may be justification for the introduction of clinical osteosarcoma screening programmes in these breeds. There is also the potential for the breeds reported at highest risk to carry a high-effect genetic mutation which, if identified, could be useful for genetic screening. A familial risk study recently suggested that a single autosomal risk factor may explain heritable osteosarcoma risk in Scottish Deerhounds, although this factor is not yet fully characterised [52]. Genetic exploration of osteosarcoma risk in other breeds is also underway [53, 74–76]. An alternative preventative strategy is to consider the extreme body size of certain breeds as itself a core predisposing risk factor [77]. Consequently, it could be possible to breed away from osteosarcoma risk by breeding away from extremes of conformation e.g. skull and leg-length or body weight, without the need for genetic knowledge or screening. This approach is in line with the concept of innate health that is promoted by the UK Brachycephalic Working Group [41].

Understanding the epidemiology of canine osteosarcoma and answering the question of “who is at risk?” is a fundamental foundation for researchers aiming to define the molecular biology of osteosarcoma. For breeds with moderate risk of osteosarcoma, where polygenic risk is considered more likely than carriage of a single high-effect risk mutation, germline genome sequencing within such at-risk breeds will likely reveal candidate genetic regions that may have functional impact when a bone cell becomes a bone cancer cell [78]. Transcriptomic and proteomic approaches may also yield such information, resulting in therapeutic advances [79–81]. Osteosarcoma is much more common in dogs than in people, meaning that there is greater availability of data for both population research and bioscience [14, 27, 82, 83] and therefore findings which benefit canine patients are likely to also benefit their human counterparts.

There are several limitations to the current analysis. The participating practices were a convenience sample and may not be fully representative of all UK veterinary practices. The quality and validity of EPR recording relied on the clinical acumen and note-taking of the veterinary professional teams. Neuter status as recorded in the originating clinical data was included in the analysis but these values inflate the likelihood of non-neutered status because the EPR might not always be updated post-neutering. The risk-factor analysis included cases that were both pre-existing as well as incident diagnoses to the 2016 study period. The statistical power for reporting breed-specific results diminished for breeds that were less common in the overall study population. For this reason, it was not possible to report reliably risk within breeds that are numerically few in the UK even where there may have been prior evidence of predisposition. Over time, as more and more practices share data with VetCompass, the statistical power to study even rarer disorders in even rarer breeds will continue to grow. Notably, some large breeds such as the Great Dane were shown to be at high risk in studies of osteosarcoma conducted 10–20 years ago, supporting the idea that a decline in ownership may have affected their rank order of breed risk in the current study [84]. Alternatively, such breeds may not be truly at risk, and improved methodology may have eradicated biases that led to them being featured previously or that changes to the breed over time may have eliminated any risk that did exist previously. As reported in the current findings and discussed, not all large breeds get osteosarcoma, and brachycephalic large breeds such as the Dogue de Bordeaux may be protected. Whilst diagnostic confirmation is nevertheless recommended, and it should not be assumed that a lytic bony lesion in an at-risk breed is always osteosarcoma, the current study, and its triangulation with histologically confirmed datasets may help to guide veterinarians in their index of suspicion, especially if further diagnostics are not feasible for economic or owner-driven reasons.

## Conclusions

The current study cements the concept that breed, bodyweight and longer leg or longer skull length are all strong risk factors for osteosarcoma in dogs. With this awareness, veterinarians can justify higher clinical suspicion of typical signs in dogs showing greater risk factors, breeders can select towards lower-risk animals by reducing giantism within breeds, and researchers can robustly define more useful study populations of predisposed breeds for fundamental and translational bioscience. The consistency of observations between the current work when triangulated across other recent analyses suggests that the findings we present here are likely to have high biological and clinical validity.

## Methods

The study population included dogs under primary veterinary care at clinics participating in the VetCompass Programme during 2016. Dogs under veterinary care were defined as those with either a) ≥ 1 electronic patient record (EPR) (free-text clinical note, treatment, or bodyweight) recorded during 2016 or b) ≥ 1 EPR recorded during both 2015 and 2017. VetCompass collates de-identified EPR data from primary-care veterinary practices in the UK for epidemiological research [38]. Data fields available to VetCompass researchers include a unique animal identifier along with species, breed, date of birth, sex, neuter status, insurance and bodyweight, and also clinical information from free-form text clinical notes, summary diagnosis terms [85] and treatment with relevant dates.

A cross-sectional analysis using cohort clinical data was used to estimate the one-year (2016) period prevalence and incidence risk of osteosarcoma and to explore associations with demographic risk factors in this population. Based on previously reported prevalence of 0.06% osteosarcoma diagnosis among insured dogs in the UK [86], power calculations estimated that at least 223,901 dogs was needed to estimate prevalence for a disorder that occurred in 0.06% of dogs with 0.01% acceptable margin of error at a 95% confidence level from a national UK population of 8 million dogs [87, 88]. Ethics approval was obtained from the RVC Social Science Ethical Review Board (reference SR2018-1652). All methods were performed in accordance with the relevant guidelines and regulations.

The case definition for an osteosarcoma case required evidence in the clinical records indicating a final diagnosis of osteosarcoma or synonym (e.g., OSA, osteosarc) for a condition that existed at any date from Jan 1, 2016 to Dec 31, 2016. Case-finding involved initial screening of all 905,552 study dogs for candidate osteosarcoma cases by searching the clinical free-text from July 1^st^ 2015 to June 30^th^ 2017 using the search terms: OS*, OSA, osteosarc*, osteosa* and osteosarc. The clinical notes of all candidate animals were manually reviewed to evaluate for case inclusion.

Breed descriptive information entered by the participating practices was cleaned and mapped to a VetCompass breed list derived and extended from the VeNom Coding breed list that included both recognised purebred breeds and also designer-crossbreed breed terms [85]. A *breed purity* variable categorised all dogs of recognisable breeds as ‘purebred’, dogs with contrived names generated from two or more purebred breed terms as ‘designer’ crossbreds (purposely bred crossbreeds) and dogs recorded as mixes of breeds but without a contrived name as ‘crossbred’ [51]. A *breed* variable included individual pure breeds and designer hybrids represented by over 5000 dogs in the overall study population or with ≥ 3 osteosarcoma cases, along with groupings of all remaining breeds and also general crossbred dogs. This approach was taken to facilitate statistical power for the individual breed analyses [89]. Breeds were also characterised by chondrodystrophy status, skull shape (dolichocephalic, mesocephalic, brachycephalic, uncategorised), and haircoat (short, medium, long, uncategorised). A *Kennel Club breed group* variable classified breeds recognised by the UK Kennel Club into their relevant breed groups (Gundog, Hound, Pastoral, Terrier, Toy, Utility and Working) and all remaining types were classified as non-Kennel Club recognised [51].

Consistent with methods previously used [89], neuter and insurance status were defined by the final available EPR value. Sex and neuter status were combined into one variable. Veterinary group described five multi-clinic veterinary groups from where the clinical data were sourced. Adult bodyweight was defined as the mean of all bodyweight values (kg) recorded for each dog after reaching 18 months old and was categorised as: < 10.0, 10.0 to < 20.0, 20.0 to < 30.0, 30.0 to < 40.0, 40.0 to < 50.0, 50.0 to < 60.0 and ≥ 60.0 or uncategorised. Mean adult bodyweight was generated for all breed/sex combinations with adult bodyweight available for at least 100 dogs in the overall study population and used to categorise individual dogs as “at or above the breed/sex mean”, “below the breed/sex mean” and “unspecified”. Age (years) was defined based on the earliest date for diagnosis of osteosarcoma in the available clinical records for cases and on December 31, 2016 (the final date in 2016 that these dogs were not a case) for non-cases. Age was categorised as: ≤ 4.0, 4.0 to < 6.0, 6.0 to < 8.0, 8.0 to < 10.0, 10.0 to < 12.0, 12.0 to < 14.0, ≥ 14.0, uncategorised.

Following internal validity checking and data cleaning in Excel (Microsoft Office Excel 2013, Microsoft Corp.), analyses were conducted using Stata Version 16 (Stata Corporation). The one-year period prevalence with 95% confidence intervals (CI) described the probability of osteosarcoma at any point during 2016. The CI estimates were derived from standard errors, based on approximation to the binomial distribution [90]. Risk factor analysis used binary logistic regression modelling to evaluate univariable associations between risk factors (*breed, chondrodystrophy, skull shape, haircoat, breed purity, Kennel Club recognised breed, Kennel Club breed group, adult bodyweight, bodyweight relative to breed-sex mean, age, sex-neuter,* and *insurance*) and osteosarcoma during 2016. Because breed was a factor of primary interest for the study, variables that derived from the breed information and therefore were highly correlated with breed (*adult bodyweight*, chondrodystrophy, *haircoat, skull shape, breed purity, Kennel Club recognised breed* and *Kennel Club breed group*) were excluded from initial breed multivariable modelling. Instead, each of these variables individually replaced the *breed* variable in the main breed-focused model to evaluate their effects after taking account of the other variables. *Adult bodyweight* (a defining characteristic of individual breeds) replaced breed and *bodyweight relative to breed/sex mean* in the final breed-focused model. Risk factors with liberal associations in univariable modelling (*P* < 0.2) were taken forward for multivariable evaluation. Model development used manual backwards stepwise elimination. Pair-wise interaction effects were evaluated for the final model variables to evaluate for biologically meaningful interactions [91]. The area under the ROC curve and the Hosmer–Lemeshow test were used to evaluate the quality of the model fit and discrimination (non-random effect model) [91, 92]. Statistical significance was set at *P* < 0.05.

## Supplementary Information

**Additional file 1: Supplementary A.** One-year (2016) period prevalence (percentage) of osteosarcoma in dog breeds with at least one case diagnosed under primary veterinary care in the VetCompass Programme in the UK. * CI confidence interval

## Data Availability

The datasets generated during and/or analysed during the current study will be made available at the RVC Research Online repository.

## Abbreviations

BMP: Bone morphogenetic protein
CI: Confidence interval
EPR: Electronic patient record
FGF: Fibroblast growth factor
GWAS: Genome-wide association study
IQR: Interquartile range
OR: Odds ratio
