# Supplementary material for: Dog breeds and conformations predisposed to osteosarcoma in the UK: a VetCompass study
Source: Canine Med Genet. 2023 Jun 27;10:8. doi: 10.1186/s40575-023-00131-2 (PMC10294386; doi:10.1186/s40575-023-00131-2)
Supplement: Supplementary file 1 — Additional file 1: Supplementary A. One-year (2016) period prevalence (percentage) of osteosarcoma in dog breeds with at least one case diagnosed under primary veterinary care in the VetCompass Programme in the UK. * CI confidence interval [file 40575_2023_131_MOESM1_ESM.docx]

## Supplementary A: One-year (2016) period prevalence (percentage) of osteosarcoma in dog breeds with at least one case diagnosed under primary veterinary care in the VetCompass Programme in the UK. * CI confidence interval

| Breed | No. in study | No. cases | Prevalence % | 95% CI* |
| --- | --- | --- | --- | --- |
| Scottish Deerhound | 122 | 4 | 3.28 | 0.90-8.18 |
| Leonberger | 270 | 4 | 1.48 | 0.41-3.75 |
| Great Dane | 1269 | 11 | 0.87 | 0.43-1.55 |
| Rottweiler | 7284 | 61 | 0.84 | 0.64-1.07 |
| Greyhound | 5456 | 34 | 0.62 | 0.43-0.87 |
| Old English Sheepdog | 737 | 3 | 0.41 | 0.08-1.18 |
| Saint Bernard | 597 | 2 | 0.34 | 0.04-1.20 |
| Bull Mastiff | 1624 | 4 | 0.25 | 0.07-0.63 |
| Newfoundland | 877 | 2 | 0.23 | 0.03-0.82 |
| German Pointer | 1925 | 4 | 0.21 | 0.06-0.53 |
| Standard Doberman Pinscher | 2461 | 5 | 0.20 | 0.07-0.47 |
| Japanese Inu Akita | 1570 | 2 | 0.13 | 0.02-0.46 |
| Lurcher | 6022 | 6 | 0.10 | 0.04-0.22 |
| Golden Retriever | 9793 | 8 | 0.08 | 0.04-0.16 |
| Dogue de Bordeaux | 3032 | 2 | 0.07 | 0.01-0.24 |
| Labrador Retriever | 59963 | 38 | 0.06 | 0.04-0.09 |
| Boxer | 9444 | 6 | 0.06 | 0.02-0.14 |
| American Bulldog | 3224 | 2 | 0.06 | 0.01-0.22 |
| German Shepherd Dog | 21371 | 11 | 0.05 | 0.03-0.09 |
| English Springer Spaniel | 20208 | 10 | 0.05 | 0.02-0.09 |
| Chinese Shar-Pei | 3649 | 2 | 0.05 | 0.01-0.20 |
| Whippet | 4686 | 2 | 0.04 | 0.01-0.15 |
| Crossbreed | 194491 | 62 | 0.03 | 0.02-0.04 |
| Labradoodle | 7485 | 2 | 0.03 | 0.00-0.10 |
| Staffordshire Bull Terrier | 53056 | 11 | 0.02 | 0.01-0.04 |
| West Highland White Terrier | 18878 | 3 | 0.02 | 0.00-0.05 |
| English Bulldog | 8409 | 2 | 0.02 | 0.00-0.09 |
| Husky | 8565 | 2 | 0.02 | 0.00-0.08 |
| Border Collie | 24390 | 2 | 0.01 | 0.00-0.03 |
| Yorkshire Terrier | 28180 | 2 | 0.01 | 0.00-0.03 |
| English Cocker Spaniel | 33077 | 2 | 0.01 | 0.00-0.02 |
